# GM-CSF enhances tumor invasion by elevated MMP-2, -9, and -26 expression

**DOI:** 10.1002/cam4.20

**Published:** 2012-11-26

**Authors:** Claudia M Gutschalk, Archana K Yanamandra, Nina Linde, Alice Meides, Sofia Depner, Margareta M Mueller

**Affiliations:** 1 Group of Tumor and Microenvironment, German Cancer Research Center (DKFZ)Heidelberg, Germany; 2 Hamamatsu TIGA Center, BIOQUANT National Center for Tumor Diseases (NCT) University HeidelbergGermany; 3 Division of Systems Biology of Signal Transduction, German Cancer Research Center (DKFZ)Heidelberg, Germany; 4 Hochschule Furtwangen UniversityCampus Villingen-Schwenningen, Germany

**Keywords:** Colon carcinoma, GM-CSF, invasion, MMP-2, MMP-9, MMP-26

## Abstract

Granulocyte–macrophage colony-stimulating factor (GM-CSF) promotes tumor progression in different tumor models in an autocrine and paracrine manner. However, at the same time GM-CSF is used in cancer therapies to ameliorate neutropenia. We have previously shown in GM-CSF and G-CSF expressing or negative skin or head and neck squamous cell carcinoma that GM-CSF expression is associated with a highly angiogenic and invasive tumor phenotype. To determine the functional contribution of GM-CSF to tumor invasion, we stably transfected a GM-CSF negative colon adenocarcinoma cell line HT-29 with GM-CSF or treated the same cell line with exogenous GM-CSF. While GM-CSF overexpression and treatment reduced tumor cell proliferation and tumor growth in vitro and in vivo, respectively, it contributed to tumor progression. Together with an enhanced migratory capacity in vitro, we observed a striking increase in tumor cell invasion into the surrounding tissue concomitant with the induction of an activated tumor stroma in GM-CSF overexpressing or GM-CSF treated tumors. In a complex 3D in vitro model, enhanced GM-CSF expression was associated with a discontinued basement membrane deposition that might be mediated by the increased expression and activation of MMP-2, -9, and -26. Treatment with GM-CSF blocking antibodies reversed this effect. The increased presence and activity of these tumor cell derived proteases was confirmed in vivo. Here, expression of MMP-26 protein was predominantly located in pre- and early-invasive areas suggesting MMP-26 expression as an early event in promoting GM-CSF dependent tumor invasion.

## Introduction

The hematopoietic growth factor granulocyte–macrophage colony-stimulating factor (GM-CSF) is produced by a variety of cell types, including macrophages, T lymphocytes, fibroblasts, endothelial cells, and keratinocytes following appropriate stimuli 1,2. GM-CSF was first described as factor mediating growth, differentiation, survival, and functional activities of macrophages, granulocytes, and other leukocytes 2, 3. Additionally, GM-CSF induces proliferation and migration of endothelial cells, thus contributing to angiogenesis 4, 5 and promotes keratinocyte proliferation, thereby stimulating wound healing 6, 7. As a consequence of these effects GM-CSF has been used in adjuvant tumor therapies. However, its use has to be regarded controversially. On one hand, GM-CSF is commonly used to ameliorate neutropenia, a severe adverse effect of radiation and chemotherapy regimes, with additional benefits on mucositis and wound healing 2, 6. On the other hand, GM-CSF was described as tumor promoting factor. In a number of tumor models, constitutive GM-CSF production was described, frequently together with the GM-CSF receptors 8–15. In cancer patients, elevated GM-CSF serum levels are considered as markers with high diagnostic sensitivity, especially in early non-small-cell lung carcinomas (NSCLCs) 10. In head and neck squamous cell carcinoma (HNSCC), GM-CSF expression together with vascular endothelial growth factor (VEGF) and platelet-derived growth factor AB (PDGF-AB) correlated with significantly poorer patient prognosis 14. In functional experiments, GM-CSF stimulates tumor cell growth and/or migration in vitro and in vivo 11, 12, 15–17. GM-CSF expression correlates with the metastatic capability of Lewis Lung Carcinomas and different murine tumors 18, 19 and enhances the invasive capacity of human lung cancer cells via increased expression of matrix degrading proteases 17. In colon cancer, elevated GM-CSF serum levels due to factor secreting tumors coincide with disseminated tumors and eosinophilia 20, and Tomita et al. 21 describe a GM-CSF regulated increase in tumor cell invasion via the stimulation of matrix metallo-proteinase 2 (MMP-2) and MMP-14 in HNSCC. Besides these direct effects on the tumor cells, recent studies classify GM-CSF as an activating factor for a tumor supporting stroma, indicating an additional level at which GM-CSF promotes tumor progression and metastasis 5, 9, 15, 16, 22. Most importantly, we have shown before in “naturally” GM-CSF and G-CSF expressing or negative tumor cells that the factor expression – especially the expression of GM-CSF – is associated with an enhanced invasion and metastasis in vivo 15, 16. Together, current literature and our earlier work indicate a progression promoting effect of GM-CSF and raised questions concerning the mechanisms behind a GM-CSF driven tumor progression. Therefore, we transfected GM-CSF and G-CSF negative HT-29 cells with a GM-CSF complementary DNA (cDNA) expression plasmid or the empty vector and established stable cell lines for further experiments aiming at identifying the mechanisms of GM-CSF driven tumor progression. Additionally, we treated the same factor-negative cell line with exogenous GM-CSF. We could demonstrate that, together with increased invasion and discontinued basement membrane assembly, GM-CSF expression correlates with an increase in pro- and active-MMP-2, -9 and -26. MMP overexpression was revertible by GM-CSF blocking antibodies in vitro. The enhanced proteolytic activity and an enhanced MMP-2, -9, and -26 expression that was associated with increasing GM-CSF expression, could be verified in vivo. Here, MMP-26 was predominantly present in pre- and early-invasive areas and during the onset of invasion. Taken together, our data suggest the enhanced expression of invasion associated MMPs specifically of MMP-2, -9, and -26, concomitant a stromal activation as mechanisms behind the GM-CSF driven tumor cell invasion.

## Materials and Methods

### Plasmids

The coding sequence for hGM-CSF was ligated into the multiple cloning site of the vector pZeoSV (Invitrogen, Life Technologies GmbH, Darmstadt, Germany) as described 11, 12, 15–17. As control, the pZeoSV vector without insert was used.

### Cell lines

The tumor cell line HNO97, established from HNSCC surgical specimen, and HT-29 were generous gifts of Christel Herold-Mende (University Clinics Heidelberg). HNO97 secretes GM-CSF and G-CSF, HT-29 secretes neither GM-CSF nor G-CSF, both express the receptors for both factors. Transfectants derived from HT-29 contain either the eukaryotic expression vector pZeoSV with the coding sequence for hGM-CSF (GM18C4, GM9D6, and GM9E6) or vector alone (ZB2, ZD1).

### Cell culture conditions

HNO97 and HT-29 were cultured in Dulbecco’s Modi?ed Eagle’s Medium (DMEM) (Cambrex, Charles City, Iowa) 10% fetal bovine serum (FBS), Penicillin/Streptomycin (100 U/100 *μ*g/mL) (D10), transfectant cell lines in D10 plus Zeozin (200 *μ*g/mL, Invitrogen) at 37°C, 5% CO_2_ in a humidified incubator, passaged at a split ratio of 1:10–1:15, and routinely tested negative for mycoplasma contamination as described 23.

### Conditioned media

In all, 2.5 × 10^3^ cells/cm^2^ were seeded in D10 and shifted to serum-free medium (105 *μ*L/cm^2^) after 24 h. After 96 h conditioned medium was harvested and stored at −80°C; viable cells were counted (Casy 1, Tuebingen, Germany).

### Growth curves

A total of 3 × 10^4^ cells/cm^2^ were seeded in 300 *μ*L/cm^2^ D10. Medium was shifted to DMEM 1% FBS (D1) and cells were counted in triplicate after 24 h and afterwards every 48 h up to 7 days.

### Cell migration assay

In all, 3 × 10^4^ cells/cm^2^ were seeded in six-well plates. Twenty-four hours after confluency the monolayer was scratched with a 200 *μ*L pipette tip (one scratch per well) and the lesions were marked. Medium was shifted to 210 *μ*L/cm^2^ DMEM (Cambrex) 1% FBS, Penicillin/Streptomycin (100 U/100 *μ*g/mL) (D1), or D1 containing 2 *μ*g/mL GM-CSF neutralizing antibody (#AF-215-NA, R&D Systems, Minneapolis, Minnesota) or irrelevant antibody (Mouse IgG1, #M7894, Sigma-Aldrich, Steinheim, Germany). Migration was documented by an overlay of microscopic pictures after 0 and 24 h and the migration area was determined using Cell (Olympus imaging software). The timeframe of 24 h was determined in preliminary experiments, regarding clearly detectable migration without closure of the lesion. Data shown are the mean of three independent experiments with 10 replicas (two wells with five replicas) each.

### RNA isolation and RT-PCR

RNA isoltion (RNeasy Mini kit, Qiagen, Hilden, Germany), RT (Omniscript RT Kit), and PCR (Taq RNA Polymerase, Qiagen) were performed as described 11, 12, 15, 16, 17.

GAPDH: 5′GGTGAAGGTCGGAGTCAACGGA3′, 5′GAGGGATCTCGCTCCTGGAAGA3′; pZeo – GM-CSF: 5′TCGGCCTCTGAGCTATTCC3′, 5′ACACGTTGGGTCTGATAGTG3′; GM-CSF: 5′TGGCCTGCAGCATCTCTGCA, 3′ACACGTTGGGTCTGATAGTG; GM-CSF receptor α: 5′CTTCTCTCTGACCAGCA, 3′ACATGGGTTCCTGAGTC; GM-CSF receptor *β*: 5′AATACATCGTCTCTGTTCAG, 3′TCACTCCACTCGCTCCAGAT.

### Tumorigenicity assays in vitro: organotypic cocultures (OTC)

For the preparation of dermal equivalents, a native type I rat collagen solution (2 mg/mL collagen-I, 10% Hank's, 10% FBS) was supplemented with 2.5 × 10^5^/2.5 mL dermal fibroblasts. Of this mixture, 2.5 mL were poured into membrane filter inserts (Falcon, Becton Dickinson, Heidelberg, Germany), placed in deep six-well trays (Becton Dickinson), and left to polymerize at 37°C. After 24 h, 1 × 10^6^ tumor cells were seeded on each dermal equivalent; the medium level was lowered to allow the tumor cells grow air exposed after attachment of the cells (24 h). For further information see 16. Fresh medium was added every second day (D10 plus 50 *μ*g/mL l-ascorbic-acid (Sigma), with or without 2.25 *μ*g/mL neutralizing antibody against GM-CSF (#MAB215, R&D Systems) or 5 ng/mL rhGM-CSF (Leukine [Sargramostim] Bayer HealthCare, Seattle). Conditioned media were collected weekly and stored at −80°C. For 4 weeks, two cultures per week were taken out and processed for histology and cryostat sectioning. Data shown are representative of two independent experiments with two replicas each.

### Gelatin zymography

Proteins of OTC conditioned media in 2× sample buffer (25% 0.5 mol/L Tris/HCl [pH 6.8], 20% glycerol, 10% sodium dodecyl sulfate (SDS), 0.1% bromphenol blue solution) were separated in 10% sodium dodecyl sulfate polyacrylamide gel electrophoresis (SDS-PAGE) gels containing 0.1% gelatin (porcine skin, Fluka). Proteases were renaturated (2.5% Triton X-100) and developed for 24 h at 37° (50 mmol/L Tris, 5 mmol/L CaCl_2_, 0.2 mol/L NaCl, and 0.02% Brij). Gel staining with Coomassie staining solution (0.5% Coomassie R250, 50% MeOH, 20% acetic acid) was followed by destaining (40% MeOH, 10% acetic acid). As control for MMPs, 10 mmol/L EDTA was added to renaturation and developing buffer.

### ELISA

With 2D/OTC conditioned media, Enzyme-linked immunosorbent assays (ELISAs) for hGM-CSF, hMMP-2, and hMMP-9 (R&D Systems: DGM00, DMP2F0, DMP900) were performed according to the manufacturers’ instructions. Data shown are mean of two independent experiments, tested in duplicate.

### Tumorigenicity assays in vivo: subcutaneous injection tumors and matrix inserted surface-transplantation assay

Tumor formation was assayed after subcutaneous injection of 5 × 10^6^ tumor cells in nude mice (swiss nu/nu, Charles River) without treatment or with daily intraperitoneal application of rhGM-CSF (250 ng/animal, Leukine [Sargramostim] Bayer HealthCare, Leverkusen, Gemany), rmGM-CSF (250 ng/animal, AFL415, R&D Systems), or vehicle solution (0.9% saline) for 2 weeks, starting at day 7 after tumor injection. Tumors were processed for histology and cryostat sectioning as described 11, 12, 15–17.

Surface transplants were prepared, transplanted on nude mice, and processed for cryostat sectioning with three transplants harvested weekly for 6 weeks as described 11, 12, 15–17. Data shown are representative of two independent experiments with three replicas for every time point. All procedures performed on animals were approved by the local government authorities (Regierungspraesidium Karlsruhe, Germany, AZ 35-9185.81/G-154/08).

**Table 1:** GM-CSF expression data of used cell lines and clones

Cell line/clone	ELISA (pg/mL/10^6^ cells)	PCR		
GM-CSF	GM-CSF	GM-CSF R*α*	GM-CSF R*β*
*Parental cell line*				
HT-29	0	−	+	+
*Control clones*				
HT-29 ZB2	0	−	+	+
HT-29 ZD1	0	−	+	+
*GM-CSF overexpressing clones*				
HT-29 GM18C4	60	+	+	+
HT-29 GM9D6	344	++	++	+
HT-29 GM9E6	449	+++	+	++
*GM-CSF positive cell line*				
HNO97	1260	+++	+	++

GM-CSF expression data: mRNA and protein expression of GM-CSF and the respective receptors in used cell lines and clones, determined by RT-PCR and ELISA.

### Indirect immunofluorescence

Indirect immunofluorescence was performed on cryosections as described 11, 12, 15–17.

Primary antibodies: pan-cytokeratin (GP-14, Progen, Heidelberg, Gemany), mCD31 (PECAM-1, Pharmingen, Heidelberg, Gemany), Collagen-4 (rabbit polyclonal, Progen), hMMP-26 (rabbit polyclonal, Abcam, Cambridge, UK), hMMP-2, and porcine MMP-9 – crossreactive with hMMP-9 (both sheep polyclonal, generous gifts of Gillian Murphy), BrdU (Exalpha, Shirley, Massachusetts)

Pictures of three immunofluorescent stainings of three different animals for each time point were analyzed, using Cell (Olympus Imaging Software), by measuring the percentage of area for the respective signal in the region of interest – the keratin positive tumor tissue or the adjacent stroma, surrounding the tumor.

### In situ zymography

Quenched fluorescein-labeled gelatin (DQ™gelatin, pig skin, Invitrogen) was mixed (1:20) with the EnzChek® Gelatinase/Collagenase Assay Kit (Invitrogen, #E12055) and incubated on unfixed frozen sections at room temperature for 16 h. After incubation, the tissue was fixed (5 min Methanol 4°C, 2 min Acetone −20°C), stained with Hoechst, and mounted. The fluorescent signal produced by proteolytic digestion of DQ™gelatin was recognized as combined gelatinase activity (MMP-2, MMP-9, and others, e.g., MMP-26).

### Statistical analysis

A two-tailed Mann–Whitney test was performed using GraphPad Prism 4.0a (San Diego, CA). Values with *P* < 0.05 were considered significant.

## Results

### Transfection of HT-29 with a sequence encoding for hGM-CSF

Earlier experiments have demonstrated a crucial role for a coexpression of GM-CSF and its receptors in the progression and invasion of skin and HNSCC 15, 16. To investigate the functional contribution of GM-CSF to tumor growth and progression, we transfected the GM-CSF negative colon adenocarcinoma cell line HT-29 with a vector encoding for hGM-CSF or as control with the empty vector. Transfected populations were isolated and GM-CSF overexpressing cell clones identified by RT-PCR and ELISA (Table 1). For functional experiments, three clonal cell lines expressing GM-CSF (low expression: GM18C4; high expression: GM9D6 and GM9E6, as detected by ELISA) and two GM-CSF negative control transfectants (ZB2, ZD1) were selected. The presence of GM-CSF R*α* and GM-CSF R*β* in the selected tranfectant clones was comparable to the parental cell line, as confirmed by RT-PCR (Table 1). Subsequently, data shown for one cell line are representative for all cell lines with the same factor/receptor profile, unless stated otherwise.

**Figure 1:**
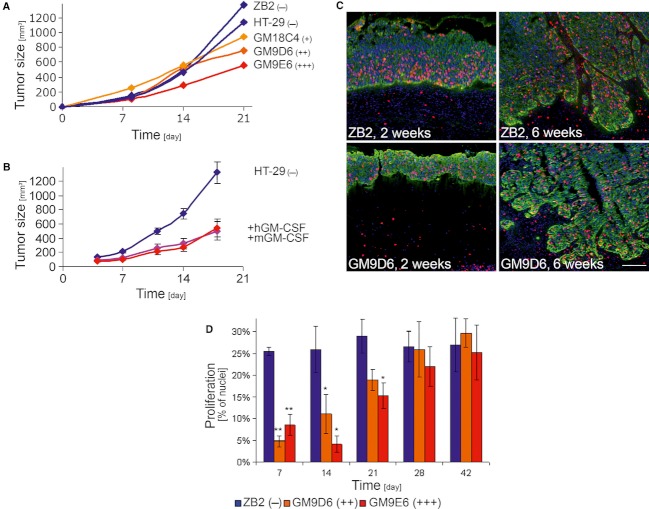
Effect of GM-CSF on tumor growth and tumor cell proliferation in vivo. (A) While tumors of GM-CSF negative cell lines (ZB2 and HT-29) reached a size of about 1200 mm^3^ after 3 weeks, injection tumors of the low GM-CSF overexpressing clone (GM18C4) reached about 900 mm^3^, and of the high GM-CSF overexpressing clones (GM9D6 and GM9E6) about 750 and 550 mm^3^, respectively. (B) And while the GM-CSF negative cell line HT-29 reached a size of 1200 mm^3^, treatment with either hGM-CSF or mGM-CSF resulted in reduced tumor growth and a final tumor volume of about 550 mm^3^ after 18 days. (C) IF staining for keratin (green), proliferation (BrdU after incorporation, red), and nuclei (blue) of nude mouse surface transplant cryosections showed a reduced number of proliferating cells in 2 weeks old surface transplants of GM-CSF overexpressing GM9D6 compared with GM-CSF negative ZB2 cells, whereas no differences were detectable after 6 weeks. (D) Quantification of tumor cell proliferation in surface transplants confirmed this, showing a constantly high proliferation rate of GM-CSF negative ZB2 transplants, compared with a significantly about 75% reduced proliferations rate at day 7 in GM-CSF overexpressing transplants, increasing for 21 days to resulting in a proliferation rate comparable to transplants of GM-CSF negative cells after 28 days. Scale bar: 100 *μ*m; *P < 0.05; **P < 0.01; d, days.

### GM-CSF overexpression results in reduced tumor cell proliferation

As the GM-CSF transfected clones coexpressed the factor with the respective receptors, we first analyzed possible autocrine effects of GM-CSF. As demonstrated by growth curves in vitro, GM-CSF overexpression induced a dose dependent reduction in proliferation compared with GM-CSF negative cell lines (Fig. S1A) in vitro. In vivo, subcutaneous injection of all cell lines in nude mice gave rise to very fast growing tumors. Again, GM-CSF overexpressing cell lines exhibited a dose dependently reduced growth rate (Fig. 1A). Moreover, treatment with either hGM-CSF or mGM-CSF resulted in the same reduction in tumor growth (Fig. 1B). The reduction in tumor cell proliferation upon GM-CSF expression was confirmed in surface transplants of the respective cell line, where tumor cells precultured on a collagen-1 gel are grafted onto the back muscle fascia of nude mice 24, The proliferation rate of GM-CSF negative control transplants as determined by immunofluorescent staining against BrdU incorporation was high at day 7 and remained constant throughout the experiment. In contrast, in GM-CSF overexpressing transplants, proliferation started at an about 75% of the control transplant and increased to reach the level of the GM-CSF negative control after 28 days (Fig. 1C and D).

### GM-CSF enhances migration, invasion, and angiogenesis

**Figure 2:**
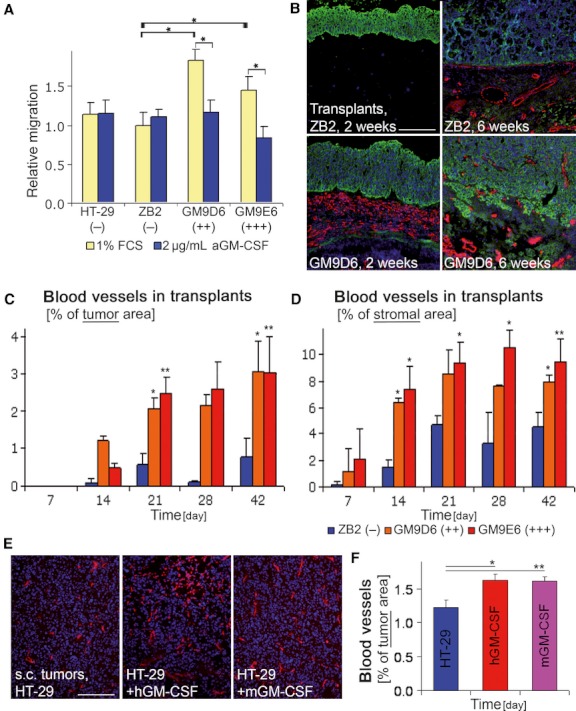
Effects of GM-CSF on tumor cell migration, invasion, and angiogenesis. (A) In a 2D scratch assay in vitro, GM-CSF overexpression (GM9D6, GM9E6) significantly enhanced tumor cell migration compared with GM-CSF negative cells (HT-29, ZB2; yellow bars), and GM-CSF blocking antibodies abrogated migration in all GM-CSF overexpressing cell lines significantly, but had no effect on GM-CSF negative cells (blue bars). (B) Immunofluorescent staining for keratin (green), CD31 positive blood vessels (red), and nuclei (blue) of cryosections from surface transplants (2 and 6 weeks). In GM-CSF negative ZB2 transplants, blood vessels rarely penetrated the collagen gel after 2 weeks and vessels reached up to the tumor border after 6 weeks with few vessels within the tumor mass. Transplants of GM-CSF overexpressing GM9E6 cells showed more early angiogenesis with extensive tumor invasion. (C) Quantification of blood vessels in the tumor area of surface transplants revealed an earlier and significantly elevated blood vessel number of GM-CSF overexpressing cells (GM9D6, GM9E6) compared with those of GM-CSF negative cells (ZB2). (D) Quantification of blood vessels in the stromal area of surface transplants also showed a significantly elevated blood vessel number of GM-CSF overexpressing cells (GM9D6, GM9E6) compared with those of GM-CSF negative cells (ZB2). (E) Immunofluorescent staining for CD31 positive blood vessels (red) and nuclei (blue) of cryosections from subcutaneous injection tumors and quantification (F). Blood vessels numbers are elevated in hGM-CSF and mGM-CSF treated tumors compared with the untreated control. Scale bar: 200 *μ*m; *P < 0.05; **P < 0.01; d, days.

The observed reduction in tumor growth and proliferation agrees with our earlier results for GM-CSF expressing versus GM-CSF negative tumor cell lines. This study indicated at the same time an important role of GM-CSF for tumor invasiveness: in vitro, tumor-derived and exogenous GM-CSF promoted tumor cell migration and the enhanced invasiveness of GM-CSF expressing tumor cells could be abrogated by neutralizing antibodies in a 3D model 16. To further elucidate the underlying mechanisms, we analyzed the influence of GM-CSF overexpression (GM9D6, GM9E6) on tumor cell migration and invasion. GM-CSF overexpression significantly stimulated tumor cell migration in vitro in a 2D scratch assay compared with GM-CSF negative parental and control transfected cell lines (Fig. 2A, yellow bars). GM-CSF neutralizing antibodies (2 *μ*g/mL), but not irrelevant control antibodies (not shown), led to significantly inhibited migration in all GM-CSF overexpressing cells, and had no effect on factor-negative cells (Fig. 2A, blue bars). Thus, our data clearly demonstrate an autocrine stimulatory effect of GM-CSF on cell migration. The enhanced tumor cell migration in vitro was complemented by enhanced tumor cell invasion upon GM-CSF overexpression in vivo. In surface transplants, GM-CSF negative cell lines (HT-29, ZB2) developed predominantly low-invasive tumors, whereas GM-CSF overexpressing cell lines (GM9D6, GM9E6) formed highly invasive tumors with tumor cells invading the underlying mouse tissue and stromal strands reaching up into the tumor mass (Fig. 2B).

We had previously observed an enhanced and persistent angiogenesis as a prerequisite for tumor cell invasion in human skin SCC tumor xenotransplants 22, 25. In agreement with this, vessel density, as determined by immunofluorescent staining against CD31 (endothelial cells), was enhanced in subcutaneous tumors upon GM-CSF treatment and overexpression (Fig. 2E and F, and not shown). Surface transplants revealed an accelerated and enhanced angiogenic response of GM-CSF overexpressing cell lines compared with GM-CSF negative clones (Fig. 2B–D), leading to a fourfold (42 days) higher amount of blood vessels within the tumor tissue (Fig. 2C) and fivefold (14 days) or twofold (21 days) higher amount of blood vessels in the tumor adjacent stroma (Fig. 2D). This enhanced and persistent stromal activation in the GM-CSF overexpressing transplants is also reflected by enhanced and persistent recruitment of granulocytes into the tumor microenvironment (Fig. S1A–E).

Taken together, GM-CSF overexpression and treatment are linked to an increased tumor cell migration in vitro and enhanced tumor cell invasion into the host stroma together with stromal activation in vivo.

### Discontinued basement membrane and enhanced MMP-2, -9, and -26 expression with increasing GM-CSF expression in a 3D organotypic model in vitro

**Figure 3:**
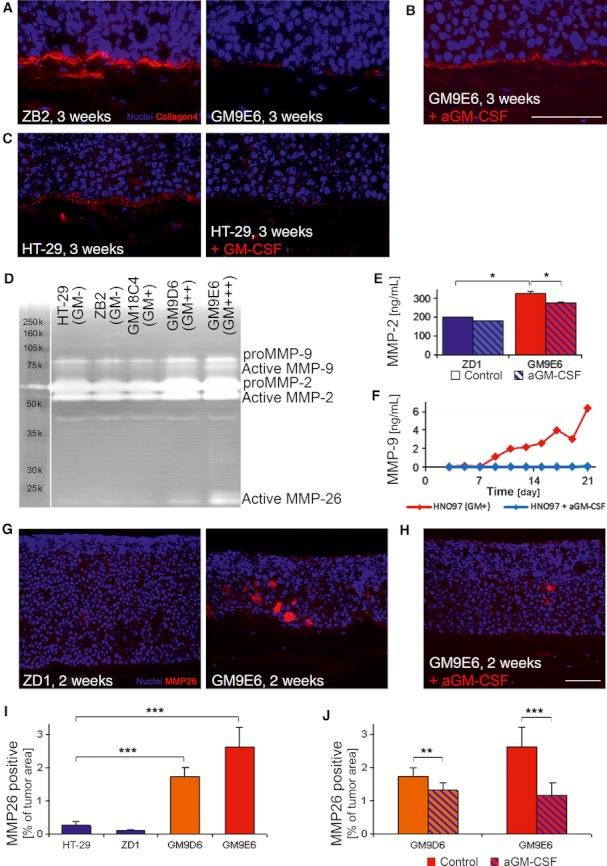
Tumor cell invasion and MMP expression in a 3D in vitro model. (A–C) Immunofluorescent staining for collagen-4 (red) and nuclei (blue) of 3D in vitro OTC cryosections (3 weeks). (A) The collagen-4 deposition at the tumor-stroma boarder decreased with increasing GM-CSF expression in the 3D in vitro OTC model. (B) GM-CSF blocking antibodies restored this collagen-4 deposition. (C) GM-CSF treatment also resulted in a decreased deposition of collagen-4 at the tumor-stroma boarder. (D) Gelatin zymographies of OTC conditioned medium revealed at day 14 an increase in pro- and active MMP-2 and MMP-9 as well as a doubled band from about 18–22 kDa that is most likely active MMP-26. (E) Significantly elevated MMP-2 secretion was verified via protein ELISA of OTC conditioned medium, as shown for GM-CSF negative (ZD1) and GM-CSF overexpressing (GM9E6) cells (unicolored bars). Moreover, GM-CSF blocking antibodies significantly inhibited MMP-2 secretion of GM-CSF overexpressing (GM9E6) but not of GM-CSF negative (ZD1) cells (striped bars). (F) For the high GM-CSF expressing HNSCC cell line HNO97, blockade with GM-CSF neutralizing antibodies resulted in complete abrogation of MMP-9, as measured by ELISA. (G) Immunofluorescent staining for MMP-26 (red) and nuclei (blue) of 3D in vitro OTC cryosections showed a strong increased cellular MMP-26 staining of GM-CSF overexpressing (GM9E6) compared with GM-CSF negative (ZD1) cells at day 14. (H) GM-CSF blocking antibodies almost abrogated MMP-26 signals of 3D in vitro OTC sections of GM-CSF overexpressing cells (GM9E6). (I) Quantification of MMP-26 staining revealed a significant increase with GM-CSF overexpression (GM-CSF negative: HT-29, ZD1; GM-CSF overexpressing: GM9D6, GM9E6). (J) GM-CSF blocking antibodies reduced this MMP-26 staining significantly in GM-CSF overexpressing clones (GM9D6, GM9E6). Scale bar: 100 *μ*m; *P < 0.05; **P < 0.01; ***P < 0.005; d, days.

To analyze the proinvasive effect of GM-CSF overexpression in more detail, in vitro experiments in our 3D organotypic culture model (OTC) 26 were performed. Tumor cells were grown air exposed on top of a collagen-1 gel containing human primary dermal fibroblasts. Cultures of all cell lines formed massive epithelia with large necrotic areas of about the same thickness (Fig. S3A). While no invasive protrusions of the tumor cells into the collagen gel or increased degradation of the collagen-1 gel could be observed (Fig. 3A, SI. 3A), staining of collagen-4 clearly revealed an increasingly discontinued basement membrane (BM) with increasing GM-CSF expression (Fig. 3A) and GM-CSF treatment (Fig. 3C), indicating an increasingly invasive phenotype with elevated GM-CSF levels. Treatment with GM-CSF blocking antibodies restored the continuous collagen-4 deposit in GM-CSF overexpressing cultures (Fig. 3B). To determine whether this observation was the consequence of enhanced collagen-4 degradation, we focused our study on the expression of MMPs that are capable of cleaving collagen-4. Gelatin zymographies and ELISA analysis of OTC conditioned media revealed enhanced expression of pro- and active- MMP-2 with increasing GM-CSF overexpression (Fig. 3D and E unicolored columns; SI. 2A). GM-CSF blocking antibodies reduced the MMP-2 expression in all overexpressing cell lines (Fig. 3E striped columns). MMP-9 expression and activation were also enhanced with increasing GM-CSF expression, as shown by gelatin zymographies of OTC conditioned media. ELISA analyses did not allow the detection of MMP-9 secretion into the conditioned media, most likely due to the detection limit (not shown). However, previously we were able to show that treatment with GM-CSF neutralizing antibodies almost abrogated tumor invasion in OTCs of a highly GM-CSF expressing HNSCC cell line (HNO97) 16 and can now demonstrate that this is associated with a decreased secretion of MMP-9 (Fig. 3F). The most striking increase in protease activity that we observed in gelatin zymographies of conditioned media of GM-CSF expressing cultures, however, was a double band of a small protease (about 18 and 19.5 kD). These bands were suppressed by EDTA treatment, validating them as MMPs (Fig S2B). In literature, different MMPs have been described with a size of approximately 18–19.5 kD and capable of cleaving collagen-4 27,28: active MMP-7 with 21 and 18 kDa 29, active MMP-12 with 45 and 22 kDa 30, and active MMP-26 with 18 kDa 31, 32. Immunofluorescence stainings against MMP-7 and MMP-12 revealed a weak signal, but no differences in the expression in control versus GM-CSF overexpressing OTCs (data not shown). However, for MMP-26, immunofluorescent stainings indicated a significantly increased cellular staining with increasing GM-CSF expression (Fig. 3G and I) that could be almost abrogated by GM-CSF blocking antibodies (Fig. 3H and J), confirming MMP-26 as the small MMP upregulated upon GM-CSF overexpression.

Thus, in our in vitro 3D OTC model GM-CSF overexpression is associated with an enhanced expression and activation of MMP-2, -9, and of MMP-26 that seems to lead to an increased degradation of the basal membrane component collagen-4.

### Protease activity and MMP-2, -9, and -26 expression in vivo

**Figure 4:**
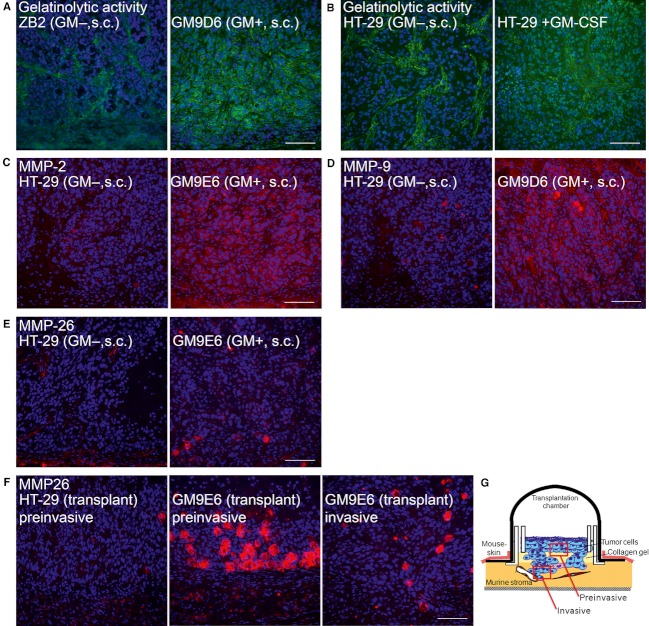
Proteolytic activity and MMP expression in vivo. (A–B) Gelatinolytic activity, as measured by in situ zymography of subcutaneous tumor cryosections, in tumors of GM-CSF negative cells (ZB2, HT-29) is located in the tumor stroma. (A) In tumors of GM-CSF overexpressing cells (GM9D6), gelatinolytic activity was also observed in the stroma, but we additionally detected a signal in the tumor cells, exceeding the stromal signal. (B) GM-CSF treatment also resulted in increased signal intensity in tumor cells. (C) Immunofluorescent (IF) staining for MMP-2 (red) and nuclei (blue) of subcutaneous tumor cryosections showed an increased staining of tumor cells in GM-CSF overexpressing (GM9E6) tumors compared with those of GM-CSF negative cells (HT-29). (D) IF staining for MMP-9 (red) and nuclei (blue) of subcutaneous tumor cryosections show, besides cellular signals in the stromal compartment, an increased staining of tumor cells in GM-CSF overexpressing (GM9D6) subcutaneous tumors compared with those of GM-CSF negative cells (HT-29). (E) IF staining of subcutaneous tumor cryosections for MMP-26 (red) and nuclei (blue) show an increased number of MMP-26 positive single tumor cells together with an increased staining of the tumor islands in GM-CSF overexpressing (GM9E6) subcutaneous tumors compared with those of GM-CSF negative cells (HT-29). (F) Moreover, IF staining of surface transplant cryosections for MMP-26 from week 4 showed a strong increased number of MMP-26 positive cells in preinvasive areas of GM-CSF overexpressing (GM9E6) compared with GM-CSF negative (HT-29) transplants. In invasive areas of the same transplants, the number of MMP-26 positive cells was downregulated again, but still exceeded the number of GM-CSF negative HT-29 transplants. (G) Schema to define the localization of “preinvasive” and “invasive” areas in surface transplants. All IF pictures depicted with the same staining are acquired under equal conditions (light intensity, exposure time, merging). Scale bar: 100 *μ*m.

To confirm the relevance of our data on the discontinuous BM and the upregulation of collagen-4 degrading MMP-2, MMP-9, and MMP-26 from the in vitro OTC model in vivo, we performed in situ zymographies of subcutaneous nude mouse tumors. GM-CSF overexpressing and GM-CSF treated tumors exhibited elevated gelatinolytic activities: In tumors of GM-CSF negative cells, gelatinolytic activity was mainly located in stromal parts, whereas tumors of GM-CSF overexpressing cells or GM-CSF treated tumors exhibited a signal for gelatinolytic activity not only in the stromal compartment but also in the tumor (Fig. 4A and B). In GM-CSF overexpressing tumors, the signal observed in the tumor compartment clearly exceeded that of the stroma (Fig. 4A). In addition, in subcutaneous tumors immunofluorescence stainings revealed an increasing signal intensity for MMP-2 and MMP-9 in the tumor islands (Fig. 4C and D) and an increasing number of distinct MMP-26 positive tumor cells (keratin positive, not shown) together with an increasing MMP-26 staining of tumor islands with GM-CSF overexpression (Fig. 4E). Interestingly, in immunofluorescent stainings of surface transplants of week 4, a strong peaking of MMP-26 was detectable in preinvasive areas and regions of starting invasion. In late transplants with established invasion, MMP-26 expression was downregulated to the level of established invasive subcutaneous tumors (Fig. 4F and G).

Thus, our in vivo data further confirm a functional contribution of GM-CSF overexpression to tumor invasion and malignancy, together with the upregulation of collagenolytic and gelatinolytic activities by enhanced expression of MMP-2, MMP-9, and MMP-26.

## Discussion

The results presented here suggest a mechanism of GM-CSF driven tumor invasion in human HT-29 tumor cells in vitro and in vivo, providing evidence that GM-CSF overexpression is correlated with an enhanced proteolytic activity and expression of MMP-2, -9, and -26 together with an increasingly disturbed BM integrity. We have previously shown a correlation of GM-CSF expression in skin and head and neck SCCs with enhanced tumor cell migration and invasion in vitro and of a coexpression of G-CSF and GM-CSF with slower, but more invasive and metastatic tumor growth, accompanied by an early and enhanced stromal activation and angiogenesis 16. Neutralization of GM-CSF in a 3D in vitro model resulted in a complete abrogation of tumor cell invasion 16. Now, we dissected the effects of GM-CSF induced invasion and stromal activation and examined the underlying mechanisms. For this purpose, we stably transfected HT-29 cells, negative for G-CSF and GM-CSF, with GM-CSF, resulting in clones of different expression levels.

GM-CSF overexpression and treatment resulted in decreased tumor growth. The diminished proliferation of HT-29 upon GM-CSF overexpression differs from the effect of GM-CSF on normal dermal keratinocytes, where it stimulates cell proliferation 7. However, these results confirm our previous observations on different HNSCC tumor cell lines, showing no effect of G-CSF and GM-CSF on proliferation in vitro and significant enhanced growth and proliferation of factor-negative tumors in vivo 16.

Despite reduced tumor growth, we could demonstrate that GM-CSF overexpression and treatment clearly contribute to tumor progression in a xenotransplant model: GM-CSF overexpressing and GM-CSF treated tumors showed a significant increase in tumor cell invasion into the surrounding tissue, concomitant with the recruitment of stromal strands into the tumor tissue and enhanced and persistent tumor angiogenesis. GM-CSF is known to act on different cells in the tumor microenvironment 4, 22, 33, 34. Thus, our findings on tumor-derived GM-CSF as stroma-activating factor are consistent with the current literature and confirm earlier findings of enhanced invasion and stromal activation, for example, recruitment of blood vessels, in GM-CSF expressing compared with negative tumors 16.

Associated with the enhanced tumor cell invasion that we observed in subcutaneous tumors together with GM-CSF overexpression or treatment, we detected a striking effect on the BM integrity in vitro. Normal epithelial cells remain separated from the underlying stroma by the BM. Disruption of the BM and invasion into the underlying tissue is one of the most important features of malignancies. Characteristics of an invasive tumor phenotype include higher levels of proteolytic enzymes and increased cell migration 35. In a 2D scratch assay, GM-CSF overexpression significantly increased tumor cell migration, as shown before for GM-CSF treatment or neutralization 16. In a complex 3D in vitro model, GM-CSF overexpression and GM-CSF treatment were associated with a decreased collagen-4 deposition, resembling a discontinued BM assembly. When analyzing matrix metallo-proteinases that are capable of cleaving collagen-4, we observed an increased expression and activation of MMP-2, -9, and MMP-26 associated with GM-CSF overexpression. GM-CSF neutralizing antibodies abolished the increase in protease expression and resulted in restored collagen-4 deposition. Enhanced expression and proteolytic activity of MMP-2, -9, and -26 upon GM-CSF were also confirmed in vivo. Interestingly, a peak of MMP-26 expression was observed in preinvasive areas, suggesting an important role in early steps of invasion.

In several tumor entities, MMP-2 and MMP-9 are described as protumorigenic MMPs, associated with lymph node metastasis and poor outcome 36, 37, and MMP-9 might also function protective in specific situations 38 Both, MMP-2 and -9, are capable of cleaving collagen-4, a major component of the BM 39, 40. Furthermore, MMP-9 and to a lesser extend MMP-2 sequester VEGF, required for the initiation of tumor angiogenesis 41.

MMP-26 upregulation is described for different tumor entities, for example, colon cancer and HNSCCs 42. It is described as epithelial-cell derived enzyme that is largely stored within the cellular compartment and only secreted in small fractions into the extracellular milieu 43. MMP-26 is capable of cleaving collagen-4, fibronectin, fibrinogen, and gelatin, and can further process proMMP-9 to the final active form 28. In vitro, MMP-26 is expressed by migrating human mucosal keratinocytes and inhibition resulted in aberrant migration and proliferation 44. In cancer, MMP-26 expression is upregulated in human breast ductal carcinoma in situ, related to invasion 45, and in prostate cancer peaking of MMP-26 and TIMP-4 marks the invasive transition 46. Other proteases and factors might be affected by GM-CSF too, but are beyond the scope of this project.

In this study, we have presented a mechanism by which GM-CSF overexpression by tumor cells as well as GM-CSF treatment induce an invasive tumor phenotype. In accordance with the literature, we could show a GM-CSF mediated increase in pro- and active- MMP-2 and -9 in vitro and in vivo. Moreover, while MMP-26 has been described in vivo in different tumor entities by others 42, 46, 47, we showed – to our knowledge – for the first time an enhanced MMP-26 expression upon GM-CSF overexpression in vivo and in a complex 3D in vitro model. There, MMP-26 protein levels peaked in pre- and early-invasive areas. Together with this enhanced MMP-26 expression upon GM-CSF overexpression, we could detect elevated levels of active MMP-9 and an increasingly discontinued collagen-4 staining in vitro. This suggests a mechanism by which MMP-26 expression in early-invasive areas leads to activation of MMP-9, upon which then is capable to process collagen-4 and VEGF and thereby drives invasion and angiogenesis.

Taken together, the data presented here describe GM-CSF as factor driving tumor progression and invasion via enhancing the expression of invasion associated MMPs, such as MMP-2, -9 and, -26, and shed some light on mechanisms of GM-CSF driven tumor progression. Moreover, the data implies MMP-26 as a potential early regulator in the onset of invasion. With regard to the therapeutic use of GM-CSF to ameliorate adverse effects of tumor therapies, further analysis of the effects of exogenous and therapeutically applied GM-CSF on the expression and activation of the described proteases in the tumor is indicated.
